# Computer-Based Identification of a Novel LIMK1/2 Inhibitor that Synergizes with Salirasib to Destabilize the Actin Cytoskeleton

**DOI:** 10.18632/oncotarget.525

**Published:** 2012-07-07

**Authors:** Efrat Mashiach-Farkash, Roni Rak, Galit Elad-Sfadia, Roni Haklai, Shmuel Carmeli, Yoel Kloog, Haim J. Wolfson

**Affiliations:** ^1^ The Blavatnik School of Computer Science, Raymond and Beverly Sackler Faculty of Exact Sciences, Tel-Aviv University, Tel Aviv, Israel; ^2^ School of Chemistry, Raymond and Beverly Sackler Faculty of Exact Sciences, Tel-Aviv University, Tel Aviv, Israel; ^3^ Department of Neurobiology, The George S. Wise Faculty of Life Sciences, Tel-Aviv University, Tel Aviv, Israel

**Keywords:** Cofilin, Ras, Rac, Rho, LIMK

## Abstract

Neurofibromin regulates cell motility via three distinct GTPase pathways acting through two different domains, the Ras GTPase-activating protein-related domain (GRD) and the pre-GRD domain. First, the GRD domain inhibits Ras-dependent changes in cell motility through the mitogen activated protein cascade. Second, it also regulates Rho-dependent (Ras-independent) changes by activating LIM kinase 2 (LIMK2), an enzyme that phosphorylates and inactivates cofilin (an actin-depolymerizing factor). Third, the pre-GRD domain acts through the Rac1 GTPase, that activate the P21 activated kinase 1 (PAK1)-LIMK1-cofilin pathway. We employed molecular modeling to identify a novel inhibitor of LIMK1/2. The active sites of an ephrin-A receptor (EphA3) and LIMK2 showed marked similarity (60%). On testing a known inhibitor of EphA3, we found that it fits to the LIMK1/2-ATP binding site and to the latter's substrate-binding pockets. We identified a similar compound, T56-LIMKi, and found that it inhibits LIMK1/2 kinase activities. It blocked the phosphorylation of cofilin which led to actin severance and inhibition of tumor cell migration, tumor cell growth, and anchorage-independent colony formation in soft agar. Because modulation of LIMK by neurofibromin is not affected by the Ras inhibitor Salirasib, we examined the combined effect of Salirasib and T56-LIMKi each of which can affect cell motility by a distinct pathway. We found that their combined action on cell proliferation and stress-fiber formation in neurofibromin-deficient cells was synergistic. We suggest that this drug combination may be developed for treatment of neurofibromatosis and cancer.

## INTRODUCTION

The actin-depolymerizing factor (ADF)/cofilin family of proteins plays a prominent role in promoting actin depolymerization [1, 2]. Cofilin is phosphorylated mainly by LIMK1 and LIMK2[3-5]. The unphosphorylated, active cofilin induces severing of actin filaments and participates in numerous cellular functions, such as cell migration, cell cycle processes, and neuronal differentiation[6, 7]. In its phosphorylated state cofilin is inactive and does not affect the cell cytoskeleton. Hyperphosphorylation of cofilin typically occurs in many human diseases and pathological conditions, such as cancer cell invasion and metastasis[8], as well as in neurodevelopmental disorders, for example Williams syndrome [9]. LIMKs are important targets in drug development because their inhibition will induce an increase in the levels of the unphosphorylated active cofilin.

**Figure 1:**
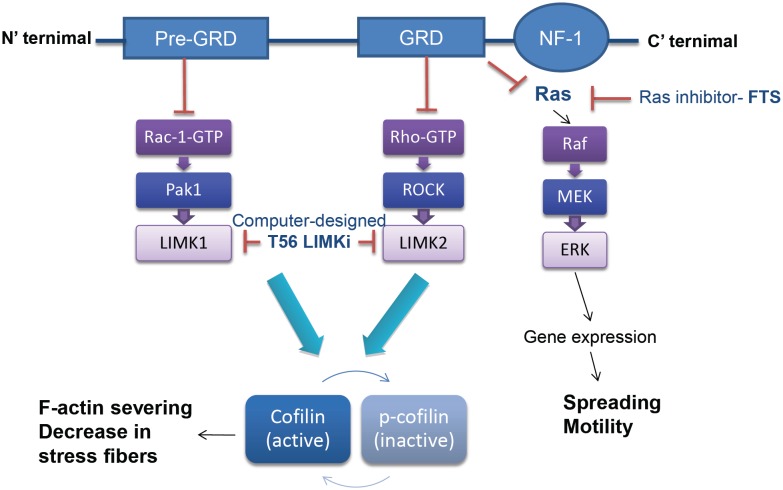
The scheme depicts the Ras-dependent and Ras-independent control of actin dynamics by neurofibromin1 The GRD domain inhibits the Ras-dependent pathway, which controls Raf-MEK-Erk-dependent gene expression and the motility and spreading of NF1^−/−^ cells. The GRD domain also inhibits a Rho-dependent pathway which activates LIMK2[2, 18]. The pre-GRD domain inhibits the Ras-independent Rac1-activation pathway, which regulates LIMK1[11]. LIMKs are widely expressed in a variety of tissues and play a critical role in regulation of the actin cytoskeleton. LIMKs phosphorylate cofilin, rendering it inactive and unable to bind and sever actin filaments.

Activation of LIMKs is regulated by the Rho GTPase family of proteins; LIMK2 is activated by the Rho GTPase pathway and LIMK1 by the Rac-1 GTPase pathway [5, 10](see Fig. 1). Recent studies have shown that p-cofilin levels are high in cells deficient in neurofibromin (NF1^−/−^ cells). These cells present relatively high levels of stress fibers [2, 11]. Neurofibromin 1, the *NF1* gene product, is a 2818-amino acid protein [12-14] containing four domains: a cysteine/serine-rich domain (CSRD), a functional Ras GTPase-activating protein (GAP)-related domain (GRD) that follows the pre-GRD domain, a leucine repeat domain, and a C-terminal domain (CTD) (see Fig. 1). The best characterized of the four is the GRD domain, which facilitates GTP hydrolysis by Ras, and exerts the major tumor-suppressor activity through its ability to downregulate the active Ras proto-oncogene and its pathways [13, 14]. The relatively high levels of active Ras.GTP that occur in NF1 deficient cells contribute to neurofibromatosis and to cancer in NF1^−/−^ patients [15]. Our group has previously shown that the high Ras.GTP phenotype of neurofibromin-deficient cells can partially be corrected by the Ras inhibitor S-trans, trans-farnesylthiosalicyclic acid (FTS; Salirasib), and that such treatment leads to the inhibition of Ras downstream effectors including MAPK, PI3K-AKT, and Ral guanine nucleotide dissociation stimulator (RalGDS). This inhibition leads in turn to reduced proliferation of NF1^−/−^ cells and tumors [16].

The GRD of nuerofibromin is known to enhance cell motility by regulating actin-filament dynamics via the Rho-ROCK-LIMK2-cofilin pathway (see Fig. 1) [1, 2, 17]. *NF1* siRNA indeed shows elevated levels of Rho. GTP, increased cofilin phosphorylation and decrease in stress-fiber formation. However, dominant-negative Ras, which itself acts upstream of Rho as well as ROCK inhibitors, suppress only partially the increased p-cofilin levels NF−/− cells. In addition, Rho activation through the GRD does not involve the classic Ras downstream pathways [2]. Those results suggested that NF1^−/−^ cell motility is controlled by additional pathways.

Recently we showed that Ras inhibition by FTS in NF1^−/−^ cells inhibits their motility and spreading, alters gene expression, and eliminates the expression of regulators of cell–matrix interaction[16]. These phenomena are indicative of a phenotypic reversion of NF1-deficient cells by FTS through inhibition of the BMP4 and TGF-β pathways [18]. Those relationships are directly related to the lack of GRD in the NF1-deficient cells [16]. However, re-expression of the entire GRD of NF1 results in only partial restoration of the excessive formation of stress fibers in neurofibromin-deficient HeLa and human fibrosarcoma cells, consistent with the notion that an additional NF1 domain or domains contribute to cytoskeleton reorganization [2].

The functional attributes of most of the neurofibromin domains other than the GRD are less well characterized than those of the GRD. Recent data support a specific role for a non-GRD domain, suggesting a Ras-independent function of NF1[3, 11]. We recently examined the possibility that the pre-GRD, N-terminal domain of neurofibromin has a regulatory function associated with remodeling of the cytoskeleton [11]. This hypothesis is supported by the non-GRD control over the cytoskeleton mentioned above, as well as by the marked evolutionary conservation of this domain. Furthermore, many missense pathogenic mutations in this domain are found in neurofibromatosis patients, suggesting that such mutations are associated with the disease, independently of the GRD [19].

We found that NF1 1-1162, the pre-GRD region of neurofibromin indeed alters the expression of genes that participate in cell adhesion and migration, and acts as a negative regulator of the Rac1/Pak1/LIMK1/cofilin pathway [5, 11, 17], a pathway highly involved in cancer [10, 20, 21] (see scheme in Fig. 1). Accordingly, in neurofibromin-deficient glioblastoma and mouse embryonic fibroblasts (MEFs), the levels of Rac1-GTP, p-Pak1, p-LIMK1 and p-cofilin are relatively high, whereas expression of the NF1 1-1163 polypeptide leads to a significant decrease in each of these active signaling enzymes [11]. Concomitantly, upon expression of the NF1 1-1163 polypeptide, actin stress fibers and focal adhesion are disassembled and cell migration is halted in neurofibromin-deficient cells [11]. These effects are independent of Ras signaling pathways. Thus, NF1 1-1163, through its negative regulation of Rac-1, shifts the balance from inactive to more active cofilin, resulting in the severing of F-actin and a decrease in actin stress fibers [11].

Altogether, these studies pointed to the possibility that neurofibromin is a participant not only in a Ras-dependent mechanism but also in a Ras-independent mechanism that regulates the actin cytoskeleton. Loss of neurofibromin would affect both pathways (Fig. 1). The newly discovered functions of NF1 [11] might be of clinical relevance for understanding the invasiveness and tumor progression of NF1-associated tumors and for drug development. In particular, it seems reasonable to target the LIMKs for cancer therapy [3]. Accordingly, based on the results of bioinformatics computational modeling, we have designed and prepared a new LIMK inhibitor.

## RESULTS

### Analysis of LIMK1/2 structures

The LIM kinases (LIMK1 and LIMK2) are dual specificity (serine/threonine and tyrosine) kinases that share 70% structural similarity in their kinase domain (3, 20).

We wanted to design an inhibitor of LIMK2 that is controlled by the best characterized NF1 domain, the GRD, which inhibits the Rho-ROCK-LIMK2-cofilin pathway (see Fig. 1). LIMK2 consists of two LIM domains, a PDZ domain, a proline/serine-rich region and a protein kinase domain. The LIM domains play an important role in regulating kinase activity [22] and their structure was solved by NMR (PDB ID: 1X6A). The PDZ domain influences nuclear-cytoplasmic shuttling [23], and its structure was also solved by NMR (PDB ID: 2YUB). The structure of the protein kinase domain of LIMK2 has yet to be solved.

### Bioinformatic identification of a LIMK inhibitor

The first LIMK inhibitor to be discovered was N-{5-(2-(2,6-dichloro-phenyl)-5-difluoromethyl-2H-pyrazol-3-yl)-thiazol-2-yl}-isobutyramide (compound 3 in Ross-Macdonald, 2008, hereafter referred to as BMS-5) [24]. It was found by screening a synthetic series of more than 400 phenyl-substituted pyrimidines [25]. BMS-5 inhibits both LIMK1 and LIMK 2 [24]. In the present study we attempted to identify an inhibitor of LIMK2 by another method. Using bioinformatic analysis to search for proteins homologous to LIMK, we identified a protein (the kinase receptor EphA3) that exhibits 31% sequence identity with it, and has a solved crystal structure, including an inhibitor, which blocks its active site. Moreover, modeling of the LIMK2 sequence on the EphA3 structure as a template revealed marked conservation of the binding-site residues.

We searched for proteins homologous to LIMK in solved structures deposited in the PDB and by using the Protein BLAST [26] and I-TASSER[27] webservers. The first homologous structure we identified was the recently solved LIMK1 (PDB ID: 3S95), which has the best sequence identity with the kinase domain of LIMK2 (64% sequence identity). LIMK1 was crystallized together with the tyrosine kinase inhibitor staurosporine. This inhibitor competes with high activity but low selectivity with ATP on binding sites of many kinases, and therefore staurosporine inhibition was not examined further.

**Figure 2:**
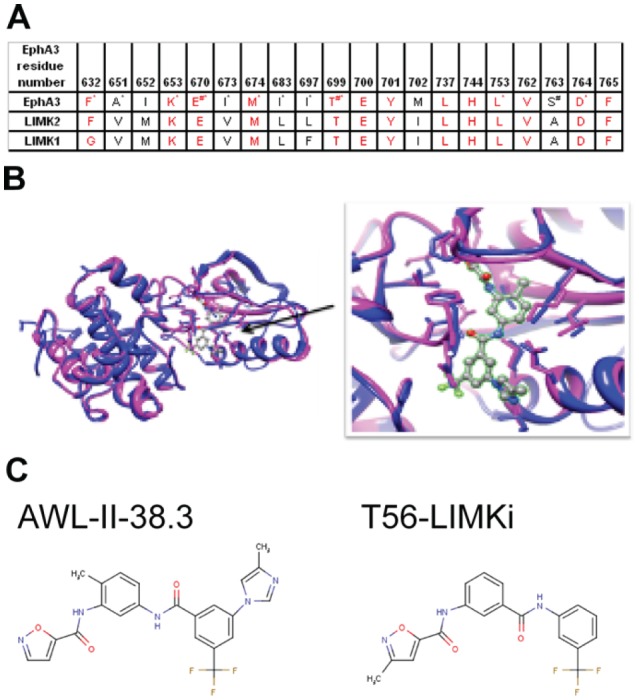
Bioinformatical analysis of EphA3 kinase and LIMK2 comparison A Binding site conservation between LIMK2 and EphA3 kinase Residues that are important for chemical interactions with the inhibitor of EphA3 are marked by:* — hydrophobic interaction; # — hydrogen bond. B Binding site conservation between EphA3 kinase (blue) and its inhibitor, AWL-II-38.3 and the modeled LIMK2 (magenta). *Left*, visualization of the whole binding domain. *Right*, focus on the AWL-II-38/3EphA3 binding site. C, *left*, EphA3 inhibitor. *right*, the similar compound T56-LIMKi, identified here as a novel LIMK 2 inhibitor.

The second LIMK homologue we identified was found, surprisingly, to be the human EphA3 kinase receptor (31% sequence identity with LIMK2). One of the PDB structures of EphA3 kinase (PDB ID: 3DZQ) was crystallized with an inhibitor termed AWL-II-38.3, which is bound in the substrate-binding pocket of the EphA3 (formula: N-(2-methyl-5-({(3-(4-methyl-1H-imidazol-1-yl)-5-(trifluoromethyl)phenyl)carbonyl} amino)phenyl) isoxazole-5-carboxamide). We applied MODELLER [28] to model the structure of the kinase domain of LIMK2 using the EphA3 kinase structure as a template and compared the inhibitor-binding sites of the two proteins. The binding site was highly conserved between EphA3 and LIMK2, suggesting to us that the EphA3 inhibitor might also inhibit LIMK2. Comparison between the binding sites of EphA3 and LIMK1 disclosed lower conservation, which might reflect a lower affinity of the inhibitor for LIMK1 than for LIMK2 (Fig. 2 A).

### Comparison between the LIMK2 and EphA3 binding sites

Comparison between the inhibitor-binding sites in EphA3 and the LIMK2 model (Fig. 2B) revealed very high conservation. Of the 20 amino acids in the binding sites, 13 showed sequence identity (65%), 6 had a different residue but the same hydrophobic property (Ala, Val, Ile and Met), and in only one amino acid (S135A) the residue differed, causing LIMK2 to lose a hydrogen bond with the inhibitor (Table 1 and Fig. 2). This high conservation supported our contention that the EphA3 inhibitor, or a similar compound, is likely to inhibit LIMK2. Our model suggested that—unlike other common kinase inhibitors which compete for the ATP binding site [29]—the compound we identified as a potential LIMK2 inhibitor occupies both the ATP-binding and the substrate-binding sites. This might provide enhanced affinity and selectivity toward LIMK2.

The binding sites of EphA3 and LIMK1 are less well conserved. The aromatic and bulky Phe632 of EphA3 is replaced by Gly346 in LIMK1, and Ile697 of EphA3 is replaced by Phe411 of LIMK1. These differences might change the shape of the binding site and reduce the affinity of the inhibitor for LIMK1 (see Fig. 2 A).

Using the ZINC database [30] we searched for commercially available compounds that are similar to the EphA3 inhibitor AWL-II-38.3. One of the compounds that most closely resembled it was T56-LIMKi. The stuctures of the two compounds are depicted in Figure 2, panel C. Upon careful analysis of the modeled LIMK2 binding site, it appeared that two additional commercially available compounds might fit into the active site of LIMK2 (MolPort-006-847-897 and MolPort-005-968-461, not shown). We next examined the effect of the T56-LIMKi compound on LIMK phosphorylation of its known substrate, cofilin, the major substrate of the LIMKs in the cells.

### T56-LIMKi reduces phosphorylated cofilin (p-cofilin) in NF1^−/−^ MEFs

**Figure 3:**
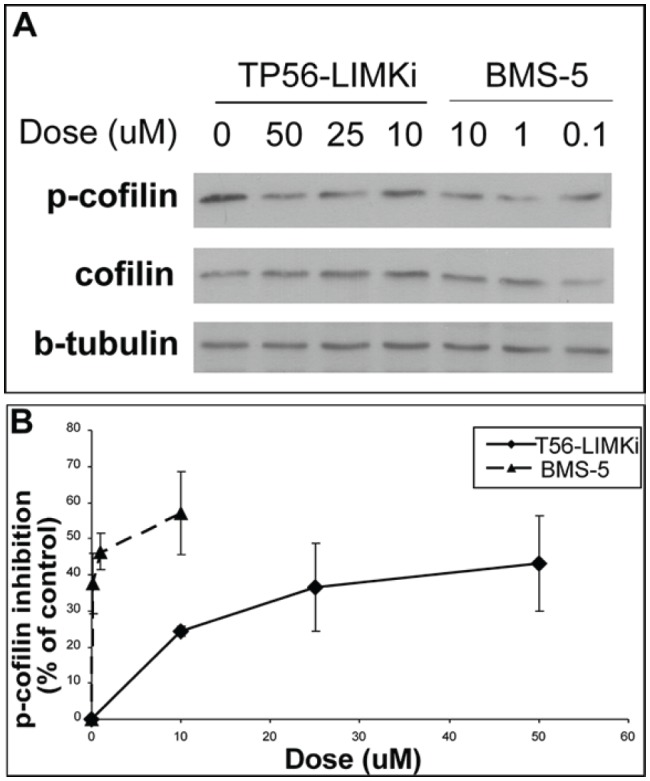
High levels of p-cofilin in NF1^−/−^ MEFs are reduced in a dose-dependent manner by T56-LIMKi Cells were serum starved for 24 hours and then treated with T56-LIMKi or BMS-5 for 2 hours at the indicated concentrations. The cells were homogenized and their proteins were immunoblotted with the specific antibody, as described in Material and Methods. A, a typical blot is presented. B, blots were quantified and normalized to beta-tubulin. Average inhibition was calculated as a percentage of control (mean ± SD, n = 3,*P < 0.05; **P < 0.01 compared to control (Student's *t*-test)).

Having previously shown that p-cofilin levels are high in NF1^−/−^ MEFs [11], we used these cells to examine the impact of T56-LIMKi on phosphorylation of the LIMK substrate cofilin. We assumed that if LIMK is inhibited by T56-LIMKi, the amount of p-cofilin in the cells will be reduced by the inhibitor. NF1^−/−^ MEFs were serum starved for 24 hours and then incubated for 2 hours with different concentrations of T56-LIMKi. The cells were lysed and subjected to immunoblotting with anti-p-cofilin, anti-cofilin, and anti-β-tubulin (loading control) antibodies (Fig. 3). We found that T56-LIMKi (10-50 μM) reduced p-cofilin in a dose-dependent manner. Notably, the inhibitor did not affect the amounts of total cofilin (Fig. 3). These results strongly suggested that T56-LIMKi inhibited LIMK, as predicted by our model (Fig. 2). A similar experiment performed with the LIMK inhibitor BMS-5 yielded comparable results, except that this inhibitor was more potent than T56-LIMKi (Fig. 3). Nonetheless these findings, taken together, strengthened the conclusion that the bioinformatic modeling procedure had predicted a novel LIMK inhibitor. These results did not distinguish, however, between the possible inhibition of LIMK 2, LIMK 1, or both by T56-LIMKi.

**Figure 4:**
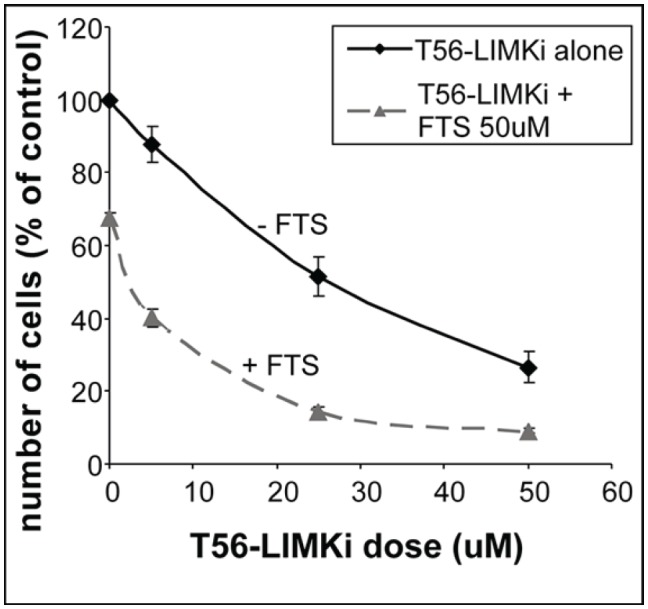
T56-LIMKi inhibits proliferation of NF1^−/−^ MEFs, and synergizes with FTS NF1^−/−^ MEFs were seeded and grown for 5 days in the absence and in the presence of the indicated concentrations of T56-LIMKi or with 0.1% DMSO (control). Cells were directly counted and typical inhibition curves are shown (means ± SEM, *n* = 9; **P < 0.01, ***P < 0.001).

Next, we examined the impact of the new LIMK inhibitor on the growth of NF1^−/−^ MEFs. The cells were plated in 24-well plates at a density of 5 × 10^3^ cells per well. Treatment of the cells with different concentrations of T56-LIMKi resulted in a dose-dependent decrease in cell number (Fig. 4). The IC_50_ of T56-LIMKi was 30 μM ± 5.3 (*n* = 9). We also examined the effects of the Ras inhibitor FTS on cell proliferation, alone and in combination with T56-LIMKi. Growth inhibition by 5 μM T56-LIMKi in the absence of FTS was only 13% ± 4.9%, but was much stronger in its presence (60% ± 2.5%; Fig. 4). Similar results were obtained with 25 μM T56-LIMKi (growth inhibition was 51% ± 2.3% in the absence of FTS and 85.5% ± 1.1% in its presence; Fig. 4). Because FTS alone caused growth inhibition of only 33% ± 1.6% (Fig. 4; zero T56-LIMKi), it seemed that the combination inhibited the growth of the NF^−/−^ cells synergistically. According to the Loewe additivity synergistic calculation [31] the combination index was 0.82, i.e., less than 1, which indicates synergism.

### T56-LIMKi and FTS induce synergistic disassembly of actin stress fibers

**Figure 5:**
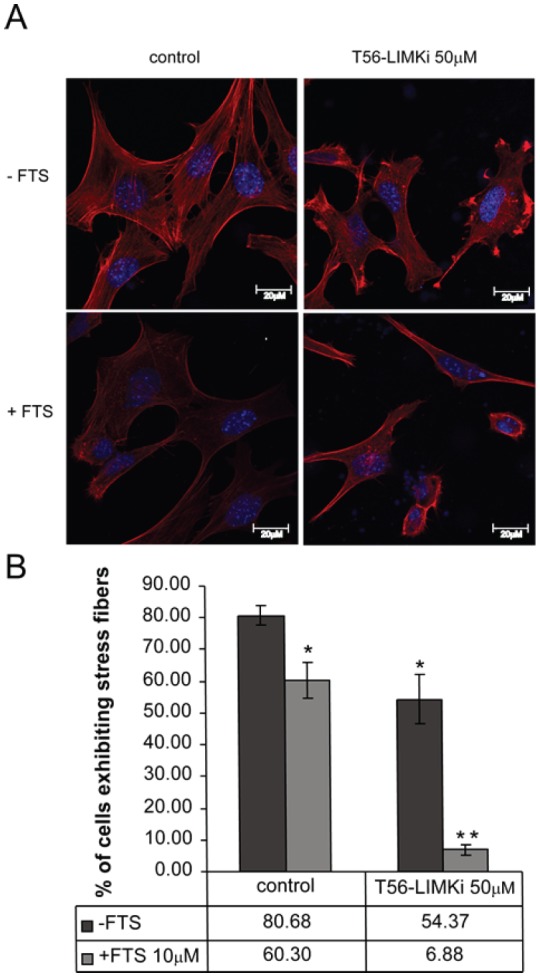
T56-LIMKi induces disassembly of actin stress fibers in NF1^−/−^ MEFs NF^−/−^ MEFs were seeded in 6-well plates at a density of 2.5 × 10^4^ cells per well. After 24 hours the medium was replaced with medium containing 0.5% FCS and the indicated amount of T56-LIMKi. After further incubation for 24 hours the cells were fixed, permeabilized, washed, and stained with phalloidin rhodamine (red fluorescence). A, typical fluorescence images are shown. B, statistical analysis of the results. The percentage of cells exhibiting stress fibers (mean ± SD, *n* = 3 slides) in a total population of 100 cells was calculated for each slide (*P < 0.05, **P < 0.01, compared to control (Student's *t*-test).

The results described above led us to investigate the effect of T56-LIMKi on the actin cytoskeleton, structures that exhibit dramatic changes during cell migration [32]. To this end we stained control (untreated) NF1^−/−^ MEFs and NF1^−/−^ MEFs treated with T56-LIMKi or FTS or their combination with rhodamine-labeled phalloidin, which labels polymeric F-actin. We then examined the effect of the T56-LIMKi alone on the cell cytoskeleton, and specifically on stress-fiber formation. Quantitative analysis of NF^−/−^ MEFs indicated that 50μM T56-LIMKi caused a statistically significant reduction in the number of cells exhibiting stress fibers (a decrease of 26% ± 7.7%; *n* = 300 cells; *P* < 0.05; Fig. 5).

Note that only 30% inhibition was obtained by the relatively high concentration of T56-LIMKi (50 μM) that inhibited 70% of cell proliferation (Fig. 4) and inhibited about 50% of cofilin phosphorylation by LIMK (Fig. 3). These results appear to support the notion that some of the LIMKs were still active. In agreement with previous results, FTS alone decreased stress fiber formation in NF^−/−^ cells by 20% ± 5.6% (Fig. 5). The decrease in stress fiber formation following the combined treatment was synergistic (74% ± 1.5%; the combination index calculated by the Loewe additive method was 0.43, i.e., less than 1, indicating synergism [31]; Fig. 5). The above results supported the notion that FTS and LIMK inhibitor operate through different pathways.

### Inhibition of cell migration by T56-LIMKi

**Figure 6:**
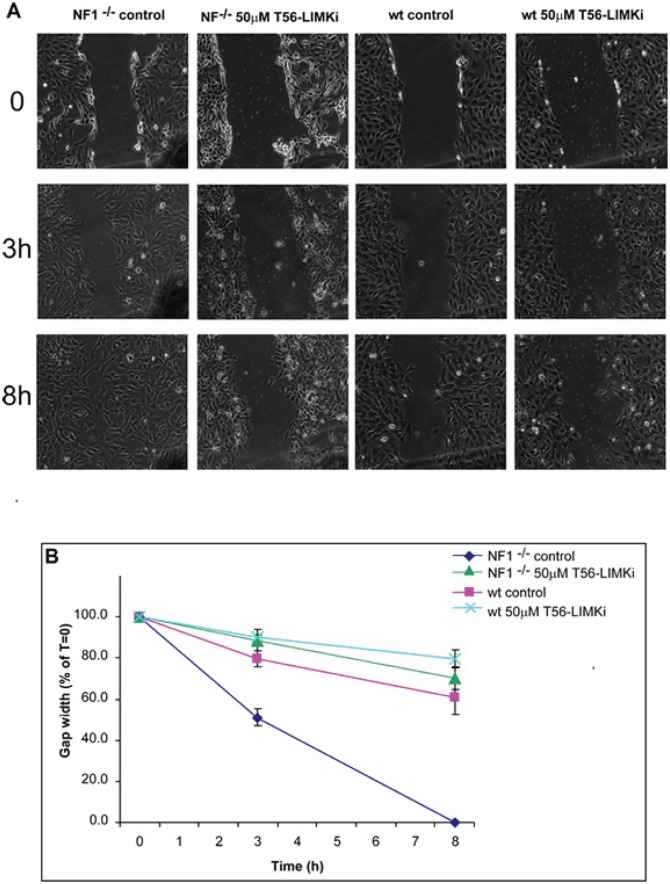
LIMK inhibition inhibits cell migration in NF1^−/−^ MEFs Wild-type and NF1^−/−^ MEFs were grown with or without 50 μM T56-LIMKi inhibitor, as described in Materials and Methods. Cells were plated on 35-mm plates at a density of 1.5 × 10^5^ cells per plate, and after 24 hours the medium was replaced with medium containing 0.5% FCS and 50 μM T56-LIMKi, or with control medium containing 0.1% DMSO. A scratch wound was inflicted, and the resulting gap was imaged at the indicated time points (hours). Experiments were performed in triplicate and measurements were obtained at three different points. A, images are from a typical experiment. B, Gap width in NF1^−/−^ MEFs (mean ± SD, *n* = 9), expressed as a percentage of the gap at the time of scratching.

To examine the effect of the inhibitor on cell migration, we performed a wound-healing cell migration assay [33] using wild-type (wt) and NF1^−/−^ MEFs. The cells were plated in 35-mm plates and incubated with or without the T56-LIMKi inhibitor. After 24 hours a scratch wound was inflicted on both sets of cells. To inhibit cell proliferation the cells were maintained in medium containing 0.5% FCS, and the width of the gap formed by the scratch was monitored at the indicated time points. Figure 6A shows the results of a typical experiment using wt and NF1^−/−^ MEFs, with and without T56-LIMKi inhibitor. The quantified results are shown in Fig. 6*B*. The gap closed faster in the untreated NF1^−/−^ MEF cells than in the treated cells (Fig. 6). For example, in the untreated cells 50% of the gap was closed within 3 hours, whereas only 10% of the gap was closed in the T56-LIMKi-treated cells (Fig. 6*A* and *B*). The mobility of the wt MEFs, unlike that of the NF1^−/−^ MEFs, was not affected by the inhibitor (Fig. 6*A* and *B*).

### LIMK inhibition decreases anchorage-independent cell growth of NF1^−/−^ MEFs

**Figure 7:**
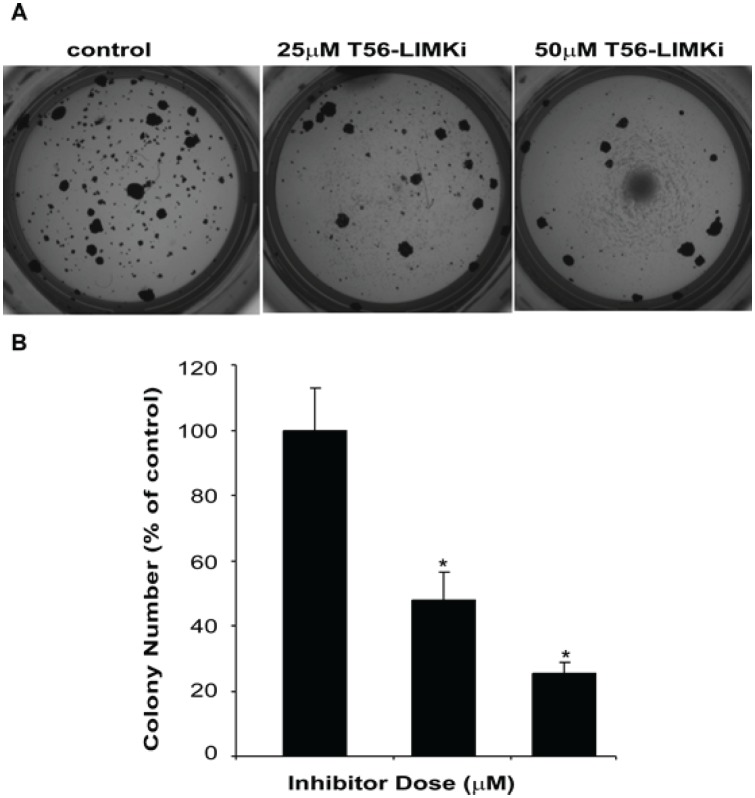
T56-LIMKi inhibits anchorage-independent growth of NF1^−/−^ MEFs NF1^−/−^ MEFs were grown in soft agar for 14 days in the absence and in the presence of the indicated concentrations of T56-LIMKi, and then stained as described in Materials and Methods. *Top*, photomicrographs of a typical experiment. *Bottom*, statistical analysis of the experiment. Columns, mean (*n* = 5); bars, SD; *P < 0.001.

As a measure of cell transformation we examined whether T56-LIMKi affects the anchorage-independent growth of the NF1^−/−^ MEFs. These cells grow in soft agar and have the ability to form colonies ([11]; Fig. 7), whereas the wt MEFs do not grow in soft agar (data not shown). We found that T56-LIMKi inhibited colony formation in a dose-dependent manner (Fig. 7).

## DISCUSSION

Neurofibromin appears to regulate cell motility by three distinct GTPase pathways through two different domains, the GRD and the pre-GRD domains (Fig. 1). The first pathway, which is controlled by the GRD domain, is the Ras-Raf-Mek-ERK pathway. This pathway is inhibited by the Ras inhibitor FTS [16]. It regulates the expression of genes that control cell spreading and cell motility [16]. The second pathway is also regulated by the GRD domain, not through classic Ras downstream effectors but rather via the Rho-ROCK-LIMK2-cofilin pathway [2]. However, in previous experiments [11], confirmed here (not shown), reduction in p-cofilin levels was not detectable by the Ras inhibitor FTS. The third pathway is regulated by the pre-GRD domain and is mediated through Rac-Pak1-LIMK1-cofilin [11, 17]. In line with previous findings, we expected that Ras would also be involved in regulation of cofilin because both Rho and Rac can be regulated by Ras (32–34). It is possible, however, that Ras affects Rho and Rac in different directions, namely, it activates Rac, which (as reported in U87 glioblastoma [34]) in turn inhibit Rho, which are deficient in neurofibromin [11]. Accordingly, it is possible that the potential regulation of cofilin by Ras is cancelled in NF1^−/−^ cells. Taken together, these data suggest that combinations of inhibitors of each of the above pathways may act synergistically to inhibit NF1^−/−^ cell spreading and motility

In a recent review of LIM kinases it was indeed suggested that these enzymes are attractive targets for drug therapy. However, only a few small-molecule modulators that are useful for drug treatment have been identified [17]. Small LIMK-inhibitor molecules have been identified by a screening of small-molecule libraries [24]. Recent studies suggested computer-based techniques for identification of new LIMK inhibitors [3, 17]. Here we employed a new computer-based procedure in which we identified the new inhibitor by searching for structural homologues of LIMK, such as EphA3, which has a known inhibitor. Following homology modeling, we first found that the active sites of the EphA3 receptor and LIMK2 are highly similar. We then used the structure of the known EphA3 inhibitor, AWL-II-38.3, and found that it fits to LIMK2 and LIMK1 ATP-binding and substrate-binding pockets (Fig. 2, C). We therefore decided to use a similar compound (T56-LIMKi), which was expected to bind with LIMK2/1 (Fig. 2, C) and block their kinase activity.

Next, we tested the compound T56-LIMKi biologically and found that it is indeed a novel inhibitor of LIMK. T56-LIMKi inhibited the phosphorylation of cofilin (see Fig. 3). Notably, the reduction in p-cofilin was accompanied by actin severing (Fig. 5) and inhibition of cell migration (Fig. 6), cell proliferation (Fig. 4), and anchorage-independent colony formation in soft agar (Fig. 7). Because LIMK1/2 regulation by NF1 is Ras-independent [2, 11, 17, 18], and because we observed a synergistic inhibition of NF1-deficient cell proliferation and stress-fiber formation by combined treatment with T56-LIMKi and the Ras inhibitor FTS (Fig. 5), we propose this combination as a novel approach of potential value for NF1 therapy. It is important to bear in mind that Ras regulates additional pathways including the MAPK-ERK, the PI3K, and the RalGDS pathways, all of which control cycle control, apoptosis, and proliferation[35] and are inhibited by FTS [36, 37]. Inhibition of these pathways, together with inhibition of the LIMKs by T56-LIMKi or other inhibitors, may also synergistically reverse the malignant phenotype of neurofibromin-depleted cells, including NF1^−/−^ schwannoma in neurofibromatosis, and of other cancer cells. The present study thus offers a possible new method for the treatment of neurofibromatosis patients

## MATERIALS AND METHODS

### Bioinformatics

Homologous proteins were identified by the Protein BLAST [26] and the I-TASSER [27] webservers. Homology modeling was performed by MODELLER [28]. We used the ZINC database [30] to search for a commercially available compound that might inhibit LIMK2. (For more details see the Results section.)

### Cell culture procedures and materials

Mouse embryonic fibroblasts (MEFs), both wild-type and NF1 knockout (NF1^−/−^), were prepared from NF1^+/−^ mice, as described previously [38]. Cells were grown in DMEM containing 10% fetal calf serum (FCS), 2 mM L-glutamine, 100 units/mL penicillin, and 100 μg/mL streptomycin. The cells were incubated at 37°C in a humidified atmosphere of 95% air and 5% CO^2^.

Compound T5601640 (defined here as T56-LIMKi) was purchased from Ambinter (Paris, France). The LIMK inhibitor BMS-5 (Bristol-Myers Squibb) was purchased from SynKinase (Shanghai, China).

### Anchorage-independent cell proliferation in soft agar

Noble agar 2% (Difco, Detroit, MI) was mixed with medium (DMEM × 2, containing 10% FCS, 4 mM L-glutamine, 200 units/mL penicillin, and 0.2 mg/mL streptomycin). The mixture (50 μL) was poured into each well of 96-well plates to provide the base agar at a final agar concentration of 1%. Agar (0.6%) was mixed with DMEM × 2, containing cells at a density that provided 8 × 10^4^ cells per well, and 50 μL of the mixture was plated on the base agar (at a final concentration of 0.3%). T56-LIMKi was prepared in DMEM × 1 containing 5% FCS at different concentrations, and 100 μL of the mixture was placed in each well so that the final concentrations of T56-LIMKi were 0, 25 or 50 μM per well. The cells were incubated for 14 days and then stained for 4 hours with 1 mg/mL 3-(4,5-dimethylthiazol-2-yl)-2 5-diphenyltetrazolium bromide (MTT; Sigma-Aldrich, St. Louis, MO), which stains active mitochondria in living cells, and the colonies were imaged. Colonies larger than 0.16 mm^2^ (mean ± SD, *n* = 5) were counted using Image-Pro Plus software (Media Cybernetics, Carlsbad, CA). The average percentage of colonies in each group (means ± SD, *n* = 5) was calculated by dividing the number of colonies of a particular treatment and specific group size by the number of colonies of the same size in the corresponding untreated control group.

### Scratch-induced migration assay

This was done as described elsewhere [34]. NF1-knockout and wt MEFs were seeded on 35-mm plates at a density of 1.5 × 10^5^ per plate. After 24 hours the medium was replaced with 0.5% FCS containing DMEM, and the cells were treated for 24 hours with T56-LIMKi (50 μM). In each plate three areas were scratched, creating three gaps of similar widths. The media and the inhibitors were then replenished. Immediately thereafter, and at the time points indicated in Results, phase-contrast images of the plates were obtained with a CCD camera connected to an Olympus fluorescence microscope (×10 objective). We marked the region imaged at zero time to enable us to photograph the same area at different times, so that we could examine a specific population of migrating cells. The widths of gaps treated with the inhibitor and at different time points were measured with the aid of Image-Pro Plus software. The data acquired from the three scratches on each plate were averaged to obtain the mean gap width at a given time. Statistical analysis disclosed either the mean gap width (in arbitrary units) of T56-LIMKi-treated cells relative to the control at different time points (means ± SD, *n* = 9) or the percentage of migration, calculated as the width of the gap still open at the final time point, expressed as a percentage of the gap size at zero time for each treatment (means ± SD, *n* = 9).

### Western blot analysis

NF1^−/−^ MEFs were plated at a density of 1× 105 or 5 × 105 cells in 6-well plates or 10-cm dishes, respectively, and were allowed to grow overnight in medium containing 10% FCS. The medium was then replaced with medium containing 0.5% FCS, and the cells were treated for 2 hours with the indicated doses of T56-LIMKi. We then lysed the cells with solubilization buffer (50 mMTris-HCl (pH 7.6), 20 mM MgCl2, 200 mM NaCl, 0.5% NP40, 1 mM Dithiothreitol, and protease inhibitors), and the lysate (50 μg) was subjected to SDS-PAGE and then immunoblotted with one of the following antibodies: anti-p-cofilin (1:1000), anti-cofilin (1:1000), anti-β-tubulin (1:500). The immunoblots were exposed to peroxidase-goat anti-rabbit IgG (1:2500), and protein bands were visualized by enhanced chemiluminescence and quantified by densitometry (EZ-Qant). Rabbit anti-cofilin and p-cofilin (Ser3) were from Cell Signaling Technolgy (Beverly, MA); mouse anti-β-tubulin antibody was from Sigma-Aldrich; peroxidase-goat anti-mouse IgG and peroxidase-goat anti-rabbit IgG were from Jackson ImmunoResearch Laboratories (West Grove, PA).

### Fluorescence staining and confocal microscopy

MEFs were seeded on glass coverslips in 6-well plates at densities of 2.5 × 10^4^ cells per well. After 24 hours the medium was replaced with medium containing 0.5% FCS and the indicated doses of T56-LIMKi. Cells were incubated for a further 24 hours and were then fixed, permeabilized, and washed. Rhodamine-labeled phalloidin was added for 30 minutes and the slides were then washed, mounted, and imaged. F-actin was visualized and then photographed under an LSM510 confocal microscope (×63 objective) fitted with rhodamine filters. For statistical analysis we counted 100 cells from each slide, with or without stress fibers, under an Olympus fluorescence microscope. Cells exhibiting stress fibers were expressed as percentages {mean ± SD) of 100 cells counted from each slide.

## Abbreviations

ADF: actin-depolymerizing factor
CSRD: cysteine/serine-rich domain
CTD: C-terminal domain
FCS: fetal calf se-rum
FTS, S-trans: trans-farnesyl thiosalicyclic acid
GAP: GTPase activating protein
GRD: GAP-related domain
LIMK: LIM kinase
MEF: Mouse embryonic fibroblast
NF1: neurofibromin
NF1−/−: neurofibromin deficient
Pak1: p21-activated kinase 1
PI3K: phospatidylinositol 3-kinase
